# Improving Orthostatic Hypotension Screening in Fall Prevention: A Two-Cycle Audit on Falls Assessment and Lying and Standing Blood Pressure Measurement

**DOI:** 10.7759/cureus.81138

**Published:** 2025-03-25

**Authors:** Yusuf Yusuf, Noor Alfardan, Ebrahim Matar, Farah Najeeb, Zainab Abdulmotaleb

**Affiliations:** 1 Trauma and Orthopaedics, Harrogate District Hospital, Harrogate, GBR; 2 General Medicine, Ministry of Health, Manama, BHR; 3 General Surgery, Antrim Area Hospital, Northern Ireland, Antrim, GBR; 4 General Medicine, Altnagelvin Hospital, Londonderry, GBR

**Keywords:** clinical audit, elderly falls, falls prevention, falls risk, postural orthostatic hypotension, quality improvement study

## Abstract

Introduction: Falls in older adults (≥65 years) are a major health concern, causing significant morbidity, mortality, and healthcare costs. Orthostatic hypotension (OH), a common but often overlooked risk factor, increases fall risk due to blood pressure drops on standing. Despite the National Institute for Health and Care Excellence (NICE) guidelines recommending postural blood pressure measurement in fall assessments, adherence remains inconsistent. This audit evaluates compliance with these guidelines and aims to improve recognition of OH to enhance patient safety.

Methods: This two-cycle audit was conducted in an orthopedic rehabilitation ward in a district general hospital in North East England, assessing 82 patients (41 per cycle) aged 65 and older. Adherence to NICE guidelines on fall assessment and lying and standing blood pressure (LSBP) measurement was evaluated. Data were retrospectively collected from electronic records over two two-week periods (December 2022 and March 2023). Following Cycle 1, interventions were implemented to improve compliance in Cycle 2.

Results: A total of 82 patients were included in this audit. Falls assessment completion remained high, improving slightly from 93% in Cycle 1 to 98% in Cycle 2 (*p* = 0.61). However, LSBP measurement saw a significant increase from 34% to 68% (*p* = 0.004). Among those assessed, the proportion with a systolic BP drop ≥20 mmHg rose from 36% to 46.4%. The percentage of patients sustaining fractures before admission remained similar (76% vs. 80%).

Conclusions: The initial audit revealed that 66% of eligible patients were not assessed for OH despite NICE guidelines recommending LSBP measurement. After targeted interventions, compliance significantly improved, demonstrating that clinical audits are an effective tool for enhancing adherence to fall prevention guidelines and improving patient care.

## Introduction

Falls among older adults - defined as individuals aged 65 years and older - are a major public health concern, leading to significant morbidity, mortality, and healthcare costs. Approximately one in three adults over 65, and half of those over 80, experience at least one fall annually [1]. In England, falls among those aged 65 and over result in more than 4 million hospital bed days each year, incurring an estimated cost of £2 billion to the National Health Service (NHS) [2]. Beyond financial implications, falls can lead to fractures, head trauma, loss of independence, and significantly reduced quality of life.

A variety of risk factors contribute to falls in older adults, including gait and balance impairments, visual deficits, cognitive decline, polypharmacy, chronic conditions affecting mobility, and age-related physiological changes affecting overall stability. One critical but frequently overlooked factor is orthostatic hypotension (OH) - a condition characterized by a sustained drop in systolic blood pressure of ≥20 mmHg or diastolic blood pressure of ≥10 mmHg within three minutes of standing [3]. OH is a common cardiovascular disorder among older adults, often resulting from autonomic dysfunction, medication side effects, or underlying chronic diseases. Its prevalence increases with age due to physiological changes, such as impaired baroreflex sensitivity and decreased vascular compliance, affecting blood pressure regulation [4]. Studies have shown that OH affects approximately 20% of community-dwelling older adults and up to 30% of those in long-term care settings [5]. It is strongly associated with dizziness, syncope, and an increased risk of falls, fractures, and mortality [6,7]. In the UK, the reported prevalence of postural hypotension ranges from 28% in older women to as high as 81% in older adults when assessed using continuous blood pressure monitoring [8].

The National Institute for Health and Care Excellence (NICE) guidelines emphasize the importance of a multifactorial fall risk assessment, which aims to identify an individual's risk factors for falling. This comprehensive assessment includes a detailed history of previous falls, evaluations of mobility and balance, cognitive function, visual impairment, urinary incontinence, medication review, and assessment of fear of falling and perceived functional ability [9]. Despite these recommendations, adherence to guidelines-particularly regarding postural blood pressure measurement - remains inconsistent, leading to missed opportunities for prevention and early intervention.

This clinical audit aims to evaluate the extent to which postural blood pressure measurements are performed in elderly patients presenting with falls, assess the implementation of multifactorial risk assessments, and propose strategies to enhance compliance with NICE guidelines. By improving recognition and management of OH and other fall risk factors, this study seeks to contribute to fall prevention efforts and ultimately enhance patient safety and outcomes.

## Materials and methods

This study was conducted in two cycles at an orthopedic rehabilitation ward in a district general hospital in the United Kingdom. This ward facilitates patient transition between acute care and discharge home. The study population included 41 patients in each cycle (total 82), all aged 65 years or older, admitted following a fall prior to their transfer from the acute hospital setting. The audit assessed adherence to two key standards: completion of a fall assessment and measurement of lying and standing blood pressure (LSBP). These standards, aligned with established guidelines and recommendations by NICE [9], aim for 100% compliance to ensure a comprehensive assessment of fall risk.

Data were collected retrospectively from the hospital’s electronic patient record system over two separate two-week periods: The first cycle in December 2022 and the second cycle in March 2023.

The two key variables assessed were: Falls Assessment: A standardized form filled out by nursing staff upon admission, documenting the patient’s fall risk level (low, medium, or high), baseline mobility, and cognitive status, and ensuring that LSBP measurements are taken. LSBP Measurement: Documentation of postural blood pressure readings to assess for OH as a contributing factor to falls.

Between Cycle 1 and Cycle 2, interventions were implemented to improve adherence to the assessment standards. These included presenting the Cycle 1 audit results during departmental teaching sessions, incorporating daily reminders in the morning huddle, distributing educational flyers, and providing targeted education to all doctors, nurses, and healthcare staff.

## Results

A total of 82 patients aged 65 years and older, admitted to the orthopedic rehabilitation ward following a fall, were included in this two-cycle audit. Adherence to fall prevention measures improved between the two cycles (Table 1). While fall assessment completion remained consistently high, increasing marginally from 93% (38/41) in Cycle 1 to 98% (40/41) in Cycle 2 (p = 0.61), the improvement was not statistically significant. However, documentation of LSBP demonstrated a statistically significant increase from 34% (14/41) in Cycle 1 to 68% (28/41) in Cycle 2 (p = 0.004). In the first cycle, of the 14 patients who had LSBP measured, 5 (36%) showed a significant systolic blood pressure drop of 20 mmHg or more. In the second cycle, of the 28 patients, 13 (46.4%) experienced a significant systolic BP drop of 20 mmHg or more. In terms of fractures sustained (Table 1), 31/41 (76%) patients in Cycle 1 and 33/41 (80%) patients in Cycle 2 sustained fractures prior to admission. These findings indicate that the interventions between cycles effectively raised awareness and enhanced adherence to fall prevention guidelines, while also identifying a greater number of patients experiencing OH.​​​​​​​

**Table 1:** Comparison of Audit Outcomes with Lying and Standing Blood Pressure (LSBP) Measurement, Systolic Blood Pressure Drop, and Fracture Sustenance BP: blood pressure; LSBP: lying standing blood pressure; df: degree of freedom

Outcome	First Cycle (n=41)	Second Cycle (n=41)	p-value	df	Effect Size (Cramér’s V)
Falls assessment completed	38/41 (93%)	40/41 (98%)	0.61 (Chi-square), 0.62 (Fisher’s exact)	1	0.11
Lying & standing BP recorded	14/41 (34%)	28/41 (68%)	0.004 (Chi-square), 0.004 (Fisher’s exact)	1	0.34
Systolic BP drop ≥ 20 mmHg (of those with LSBP)	5/14 (36%)	13/28 (46.4%)	-	-	-
Sustained fractures (pre-admission)	31/41 (76%)	33/41 (80%)	-	-	-

## Discussion

The present audit illustrates how the process of clinical auditing, paired with staff education initiatives, can be a powerful tool in improving the measurement and documentation of LSBP for elderly patients at high risk of falls. Despite improvement, more must be done.

A robust fall risk assessment, as outlined by NICE, involves a comprehensive evaluation of various factors, including the patient's medical background, medications, functional ability, and the environment in which they reside [9]. Among the most significant contributors to falls in older adults is OH, a condition that has been shown to directly influence fall risk [10]. OH can sometimes go undetected as it often presents without symptoms, underlining the need for regular screening in high-risk populations [11].

Between the two audit cycles, there was a minor improvement in the completion of the fall risk assessment bundle by nursing staff, which included documenting the patient’s fall risk classification (low, medium, or high), baseline mobility, and cognitive status, and ensuring LSBP measurement. In the first cycle, 93% (38/41) of patients had a fall risk assessment completed, while 34% (14/41) had LSBP measured. In the second cycle, the completion of the fall risk assessment increased to 98% (40/41), and the proportion of patients receiving LSBP measurement improved to 68% (28/41), indicating a notable improvement in adherence to the protocol. In both cycles, there were patients with significant OH of at least 20 mmHg, with a higher detection rate in the second cycle due to an increase in LSBP measurements. This improvement helped identify and guide the treatment of affected patients.

Despite established guidelines, the measurement of LSBP is frequently overlooked or inconsistently documented in clinical practice. The Royal College of Physicians (RCP) emphasizes the importance of standardized training to improve LSBP measurement and documentation, but a gap remains in clinical adherence to these practices, as evidenced by the findings of this audit [12]. In our initial audit cycle, a significant proportion of patients did not undergo proper assessments for OH, and when LSBP was measured, it was often documented improperly or incompletely. One of the key challenges identified was the difficulty of conducting LSBP assessments, particularly for post-operative patients who require assistance to stand for sufficient periods to obtain accurate measurements. LSBP assessments are inherently labor-intensive, as they require patients to be recumbent for at least five minutes and then measured again at 1 and 3 minutes after standing-often requiring around 10 minutes per patient.

To address these challenges, several interventions were implemented following the first audit cycle. These included formal teaching sessions, continuous reminders during morning huddles, and the distribution of educational materials for staff members. Additionally, it was suggested that LSBP measurements could be integrated into patients’ physiotherapy sessions, where they could be taken just prior to starting the session. These measures led to a marked improvement in the second audit cycle, demonstrating the efficacy of education and continuous feedback. However, it remains essential to sustain these improvements through ongoing training and regular audits to ensure compliance over the long term.

While accurate LSBP measurement is important, preventing falls in elderly patients requires a comprehensive approach. Key strategies include the involvement of a multidisciplinary team (MDT), structured physiotherapy programs to enhance strength and safety during mobilization, thorough medication reviews with communication to the primary care team, and optimizing bone health with calcium and vitamin D supplementation-all of which help reduce the risk of falls [13-17]. Furthermore, high-risk patients should undergo an environmental fall risk assessment that considers various factors, including slippery or uneven surfaces, clutter, inadequate lighting, improper footwear, step hazards, unsafe railings, and loose mats [18-20]. Coordinated efforts between the MDT can support safe discharge and minimize fall risk.

This audit has some limitations. It only assessed whether the falls bundle was completed, not if it was done correctly, as LSBP was marked as completed in the falls assessment even when it was not. It also focused solely on LSBP documentation rather than the actual blood pressure values. Variations in patient recovery and mobility affected the timing of LSBP measurements, potentially influencing the results. While improvements in LSBP documentation were seen in the second cycle, the long-term sustainability of these changes remains uncertain without ongoing education and audits.

## Conclusions

In conclusion, this audit emphasizes the importance of clinical auditing and staff education in enhancing LSBP measurement and documentation for elderly patients at high risk of falls. While improvements were noted, sustained adherence will require ongoing education, regular auditing, and continuous reinforcement of best practices. A multidisciplinary approach is crucial to further reducing fall risk. Future efforts should prioritize the standardization of LSBP measurement procedures, the integration of automated reminders into electronic health records, and the exploration of alternative assessment methods, particularly for patients with limited mobility, to ensure comprehensive and effective fall prevention.
